# Multiomics profile and prognostic gene signature of m6A regulators in uterine corpus endometrial carcinoma

**DOI:** 10.7150/jca.46386

**Published:** 2020-09-09

**Authors:** Yizi Wang, Fang Ren, Zixuan Song, Xiaoying Wang, Xiaoxin Ma

**Affiliations:** Department of Obstetrics and Gynecology, Shengjing Hospital of China Medical University, No. 36, Sanhao Street, Heping District, Shenyang 110004, China.

**Keywords:** Uterine corpus endometrial carcinoma, m6A, TCGA, Gene signature, Prognosis

## Abstract

Uterine corpus endometrial carcinoma (UCEC) is the most common type of gynecologic malignancy worldwide. Despite advances in the treatments of UCEC, its incidence and mortality rates are still increasing. N6-methyladenosine (m6A) is the most common form of RNA modification and has attracted increasing interest in cancer pathogenesis and progression. Thus, we aimed to identify the landscape of m6A regulators and build a prognostic gene signature in UCEC. In this study, we first analyzed copy number variations (CNVs), single nucleotide variations (SNVs) and gene expression profiles as well as matched clinical information of UCEC patients from The Cancer Genome Atlas (TCGA) database. Next, we determined that CNVs in m6A regulatory genes had a significant negative impact on patient survival. The mRNA expression levels of a total of 16 m6A regulators were significantly correlated with different CNV patterns. Using univariate Cox regression analysis, IGF2BP1, KIAA1429, IGF2BP3, YTHDF3, and IGF2BP2 were found to be closely associated with UCEC patient survival outcomes. Based on the least absolute shrinkage and selection operator (LASSO) and multivariate Cox regression models, we built a 3-gene (IGF2BP3, KIAA1429 and IGF2BP1) signature of m6A regulators with prognostic value in UCEC that could effectively predict patient prognosis (log-rank test *p*-value < 0.0001). In addition, risk scores were significantly different between patients stratified by tumor stage, SNV, and CNV. Multivariate Cox regression analysis suggested that risk score might be an independent prognostic indicator for the overall survival of patients with UCEC (*p*-value < 0.05). Gene enrichment analysis indicated that high IGF2BP1 gene expression is associated with cytoplasmic stress granules. KIAA1429 gene expression is associated with cellular nucleic acid metabolism. The expression of the IGF2BP3 gene is associated with RNA binding processes. In conclusion, we determined that genetic alterations in m6A regulatory genes could be effective and reliable biomarkers for UCEC prognosis prediction.

## Introduction

Uterine corpus endometrial carcinoma (UCEC) is the sixth most common type of cancer in women and the second most common gynecologic malignancy worldwide. It was estimated that there were 382,069 new cases and 89,929 deaths in 2018 1. In addition, UCEC ranks as the second most common cancer of the female genital system 2, and the five-year overall survival rate is 55.1% in China 3. However, cancer survival in China is much lower than that in developed countries and varies substantially by geographical area 4. It also decreases with advancing stage 2. UCEC consists of two broad categories: hormone-driven type I has a good prognosis, and hormone-dependent type II has a poor prognosis 5. In the overview of the main clinical research progress on UCEC in 2018, increasing attention was paid to sentinel lymph node mapping, adjuvant therapy and targeted therapy 6. Despite advances in the treatments of UCEC, the incidence and mortality rates are still increasing 7. Thus, to improve the survival outcomes of UCEC patients, it is important to identify novel clinical potential prognostic biomarkers and therapeutic targets.

RNA modification, as an epigenetic change, plays significant roles in many diseases, especially cancers. N6-methyladenosine (m6A) is the most common form of RNA modification and has attracted increasing interest in recent years 8. Dynamic and reversible modification of the m6A biological process is carried out by three elements: methyltransferases (named "writers"), m6A-binding proteins (named "readers"), and demethylases (named "erasers") 9. The underlying mechanism of m6A in cancer pathogenesis and progression has been reported in various studies 10. For example, as one of the “eraser” genes, FTO is overexpressed in cervical cancer tissues and correlated with its progression. It can take part in cervical cancer cell proliferation and migration 11. This gene was also shown to promote the growth of lung cancer cells by regulating the m6A level of USP7 mRNA 12. Therefore, the molecular roles of m6A regulators in human cancers are very significant.

Recently, based on gene expression profiles from TCGA and GEO databases, researchers identified various prognostic gene signatures of human cancers, including m6A-related gene signatures. For example, Zhao *et al.* 13 used the gene expression data for head and neck squamous cell carcinoma from the TCGA database to build a two-gene prognostic signature including YTHDC2 and HNRNPC. Chen et al. combined the LASSO Cox regression model and TCGA data. They constructed a risk signature containing three m6A RNA methylation regulators including FTO, YTHDC1, and WTAP 14. In gastric cancer, three m6A RNA methylation regulators (FTO, RBM15, and ALKBH5) are not only a collective independent prognostic indicator, but they can also predict clinicopathological characteristics 15. However, there have been no studies regarding an m6A regulator gene signature in UCEC.

In this study, we first analyzed the CNV, SNV and gene expression profiles as well as matched clinical information of UCEC patients from TCGA database. We determined that the CNV in m6A regulatory genes had a significant negative impact on patient survival. The mRNA expression levels of a total of 16 m6A regulators were significantly correlated with different CNV patterns. IGF2BP1, KIAA1429, IGF2BP3, YTHDF3, and IGF2BP2 were closely associated with UCEC patient survival outcomes. Based on LASSO and multivariate Cox regression models, we built a 3-gene (IGF2BP3, KIAA1429 and IGF2BP1) signature of m6A regulators with prognostic value in UCEC that can effectively predict patient prognosis. Multivariate Cox regression analysis suggested that risk score might be an independent prognostic indicator for the overall survival of patients with UCEC. In conclusion, we determined that genetic alterations in m6A regulatory genes could be effective and reliable biomarkers for UCEC prognosis prediction.

## Materials and Methods

### Data acquisition and processing

All UCEC clinical data, CNV, SNV, and RNA-sequencing data were retrieved and downloaded from the TCGA website (https://cancergenome.nih.gov/) by TCGA-assembler 16. The download time was June 2019. We used the RTCGA R package (https://rtcga.github.io/RTCGA/index.html) for CNV download as level 3 files. For SNV data, we used the same method as CNV download, and the files were processed with MuTect 17. For the transcriptome data, we obtained a total of 555 cancer samples downloaded as read counts and normalized them via the DESeq R package. For the SNV data, we obtained data from a total of 542 cancer samples, which were downloaded as level 3 data after MuTect processing. For the CNV data, there were 558 cancer samples (level 3) with the “Segment_Mean” value. Finally, for clinical information data, there were 560 cases of UCEC. After integrating data, we excluded samples with incomplete clinical information and survival time less than 30 days. Thus, there were 515 UCEC samples with complete CNV, SNV and mRNA data of m6A-related genes in further studies (**Table S1**).

### LASSO model and signature construction

The LASSO model is an L1 regularization method, in which the L1 norm is executed to correct the weight of features in the process of building the regression model. The regularization process forces the eigenvalues to 0 and generates a sparse eigenspace. Here, three genes were selected to construct the signature, and each coefficient was obtained through a penalty procedure. A risk score formula was established as follows:

Risk score = ∑m6A gene * coefficient

This model was generated by using the glmnet package in R 18.

### Gene set enrichment

Gene set enrichment analysis (GSEA) was performed to elucidate the biological pathways of our prognostic gene signature by using software and data downloaded from the website 19. The standardized p-value of all samples, which were divided into two groups by low and high expression according to the median expression level, was < 0.05 and the false discovery rate (FDR) was < 0.25, which was considered to be extremely enriched.

### Statistical analysis

We used the R language (version 3.4.3) for all statistical analyses. Univariate Cox regression analysis was used to explore the association between CNVs and SNVs in m6A regulatory genes and clinicopathological characteristics. The difference in survival between the high-risk group and the low-risk group was calculated by the Kaplan-Meier method with a two-sided log-rank test. A receiver operating characteristic (ROC) curve was constructed to evaluate the prediction accuracy of the prognostic model. A *p*-value < 0.05 was considered significant.

## Results

### Multiomics data of m6A regulatory genes in UCEC patients

**Figure 1:**
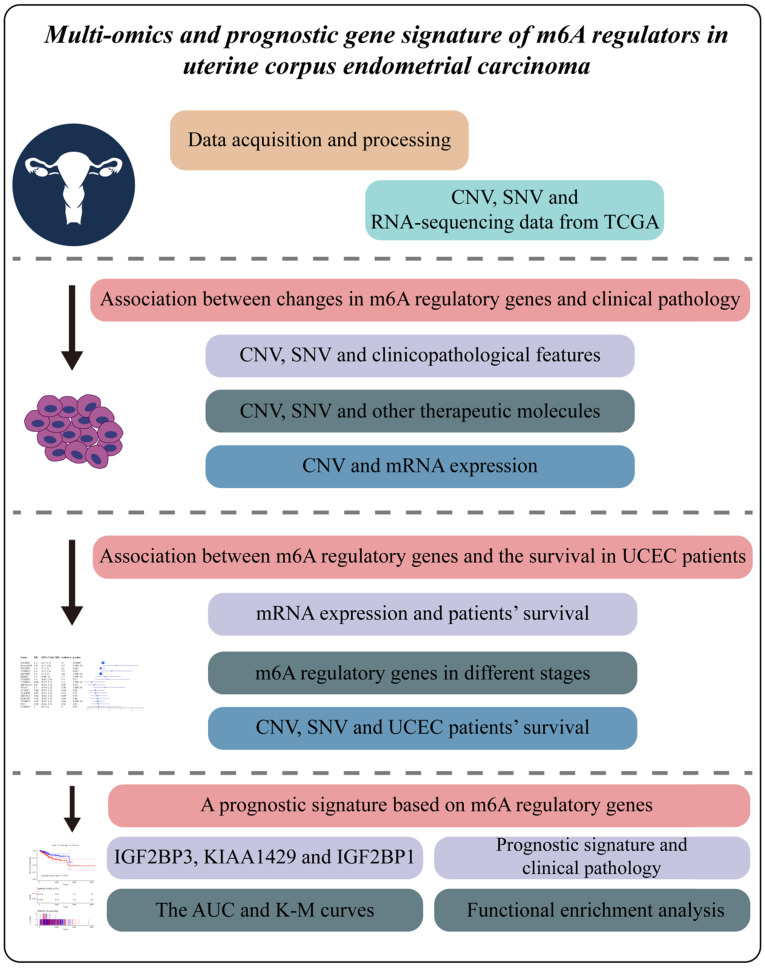
** Study workflow.** Multiomics profile and prognostic gene signature of m6A regulators in uterine corpus endometrial carcinoma.

**Figure 2:**
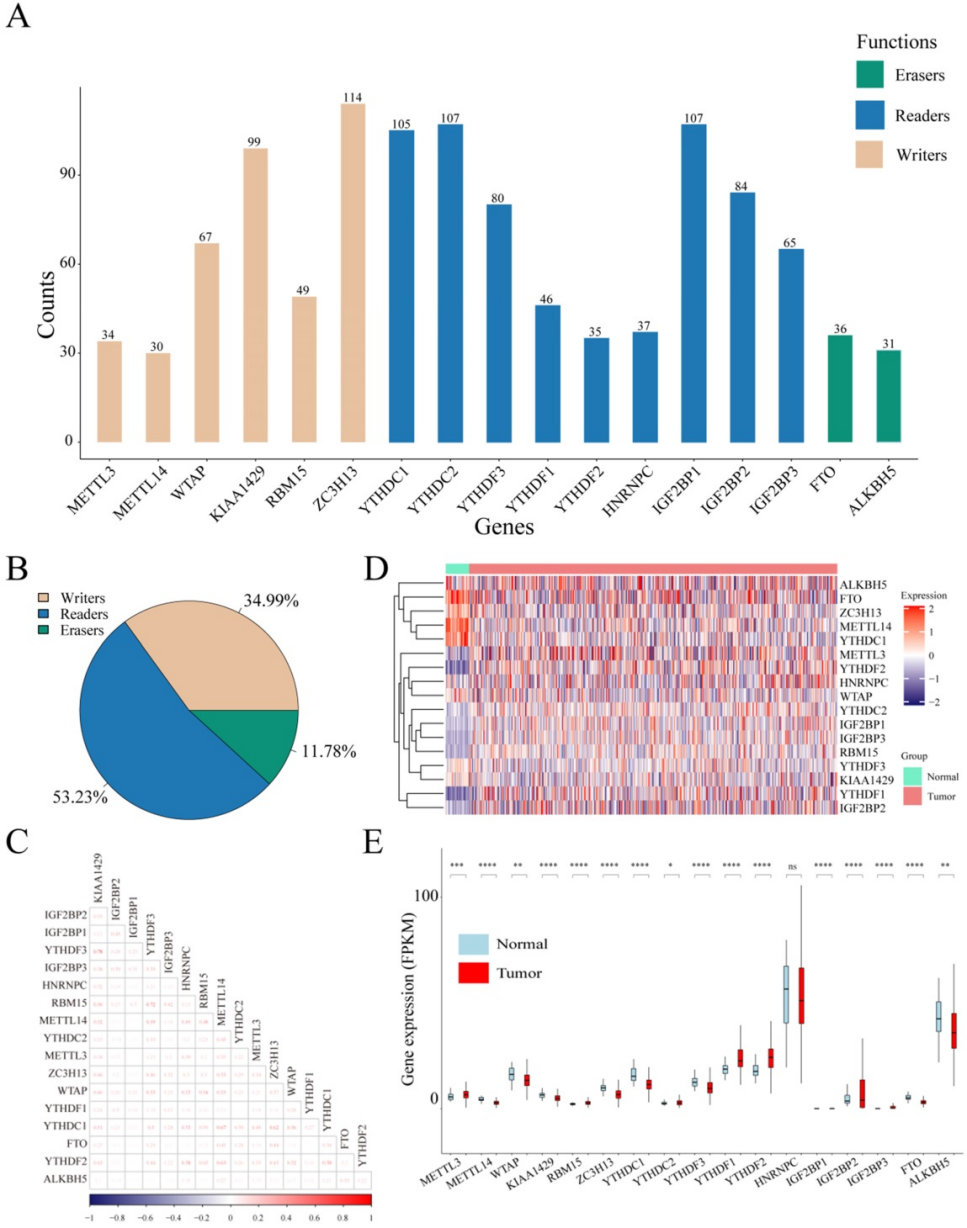
** Mutations and CNVs in m6A regulatory genes in UCEC patients.** (**A**) Frequency of mutations in different m6A regulatory genes in patients' samples. (**B**) The CNV statistics of m6A regulatory genes in samples. (**C**) The expression heatmap of m6A regulators in all UCEC samples from TCGA database. (**D**) The gene expression correlation analysis of m6A regulators. (**E**) The box plot of m6A regulators in all UCEC samples from TCGA database.

Considering the biological functions of m6A regulators in the tumorigenesis of cancer, we performed a comprehensive bioinformatic analysis of mutations, CNVs and transcriptome data, as well as the gene prognostic signature of m6A regulators in UCEC based on the TCGA database (**Figure 1**). In the SNV data of 542 tumor samples, mutations of m6A regulatory genes appeared in 200 independent samples (**Table S2**). Among them, a mutation in the "writer" gene ZC3H13 was the most frequent and was detected in 64 tumor samples with 114 mutations, accounting for 10.12% of the total m6A gene mutations. The "reader" genes have a greater frequency of mutations than the "writer" and "eraser" genes, while the "eraser" genes have a higher mutation frequency overall (**Figure 2A**). However, in the 558 UCEC samples with CNV data, the m6A regulatory genes were observed to have a high frequency of CNV events (**Figure 2B**). For example, the "writer" gene KIAA1429 has the highest frequency of CNV events of 30.87%, followed by the "reader" gene IGF2BP2 with a frequency of 28.65% and the "eraser" gene FTO with a frequency of 27.34% (**Table 1**). In addition, we identified correlations between all m6A regulators. As shown in **Figure 2C**, there was a high correlation between YTHDF3 and KIAA1429 (correlation coefficient = 0.78) and YTHDF3 and RBM15 (correlation coefficient = 0.72). Interestingly, most m6A regulator genes were differentially expressed between tumor and normal samples, except for HNRNPC (**Figure 2D and 2E**).

### Association between changes in m6A regulatory genes and clinical pathology

Next, we evaluated the relationship between changes in m6A regulatory genes (CNVs and/or mutations) and the clinicopathological features of UCEC patients. We first performed a univariate Cox regression analysis of each clinical feature. The results showed that all clinical factors were significantly associated with patient survival (*p*-value < 0.0001). In addition, the results showed that changes in m6A regulatory genes (CNV or SNV) had a significant negative impact on patient survival (HR > 1, **Table 2**). Moreover, molecular changes in the SNV and CNV of m6A regulatory genes may also be associated with changes in other therapeutic molecules in UCEC. Since PTEN, CTNNB1, PIK3CA, ARID1A, and KRAS play important roles in the pathogenesis of UCEC 20, we further evaluated whether the variation (SNV or CNV) of m6A regulatory genes was associated with changes in the above five genes. As expected, changes in m6A regulatory genes were significantly associated with changes in PTEN, CTNNB1, PIK3CA, and ARID1A. Here, only 7 of the changes in m6A regulatory genes were absent in 542 patients with CTNNB1 alterations (**Table 3**).

**Figure 3:**
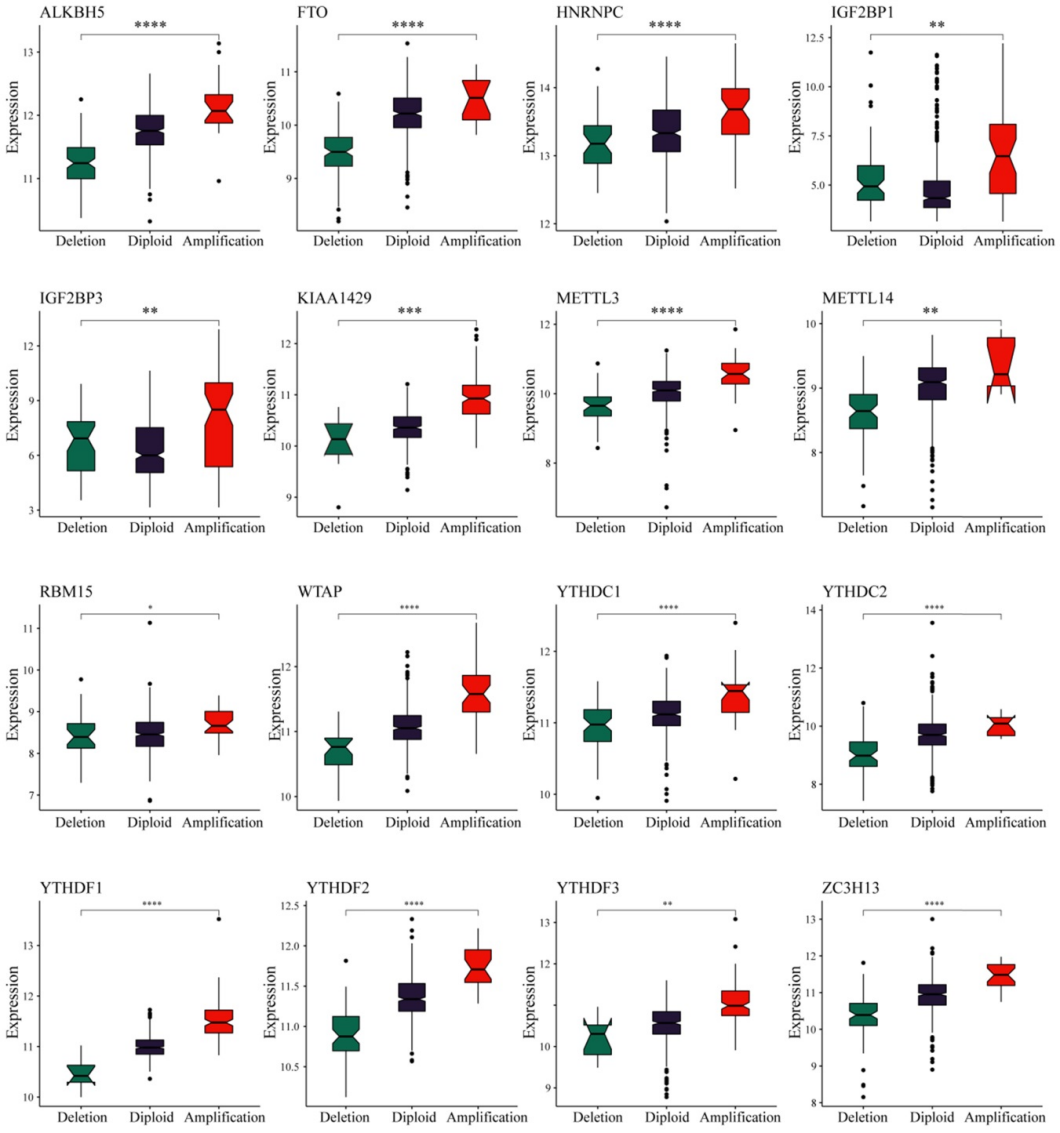
** Relationships between the CNVs and expression levels of m6A regulatory genes.** * represents a *p* value < 0.05, ** represents a *p* value < 0.01, *** represents a *p* value < 0.001, and **** represents a *p* value < 0.0001.

We observed in the previous analysis that the CNV changes of the m6A regulatory genes were significantly greater than those of SNVs. Moreover, changes in CNV can affect gene expression levels. To this end, we next evaluated the effect of m6A regulatory gene changes on mRNA expression. The results showed that mRNA expression levels were significantly correlated with different CNV patterns in 555 UCEC samples. For all 17 regulatory genes, 16 of the genes with increased copy number were associated with higher mRNA expression, whereas deletions resulted in decreased mRNA expression (**Figure 3**).

### Association between m6A regulatory genes and survival in UCEC patients

**Figure 4:**
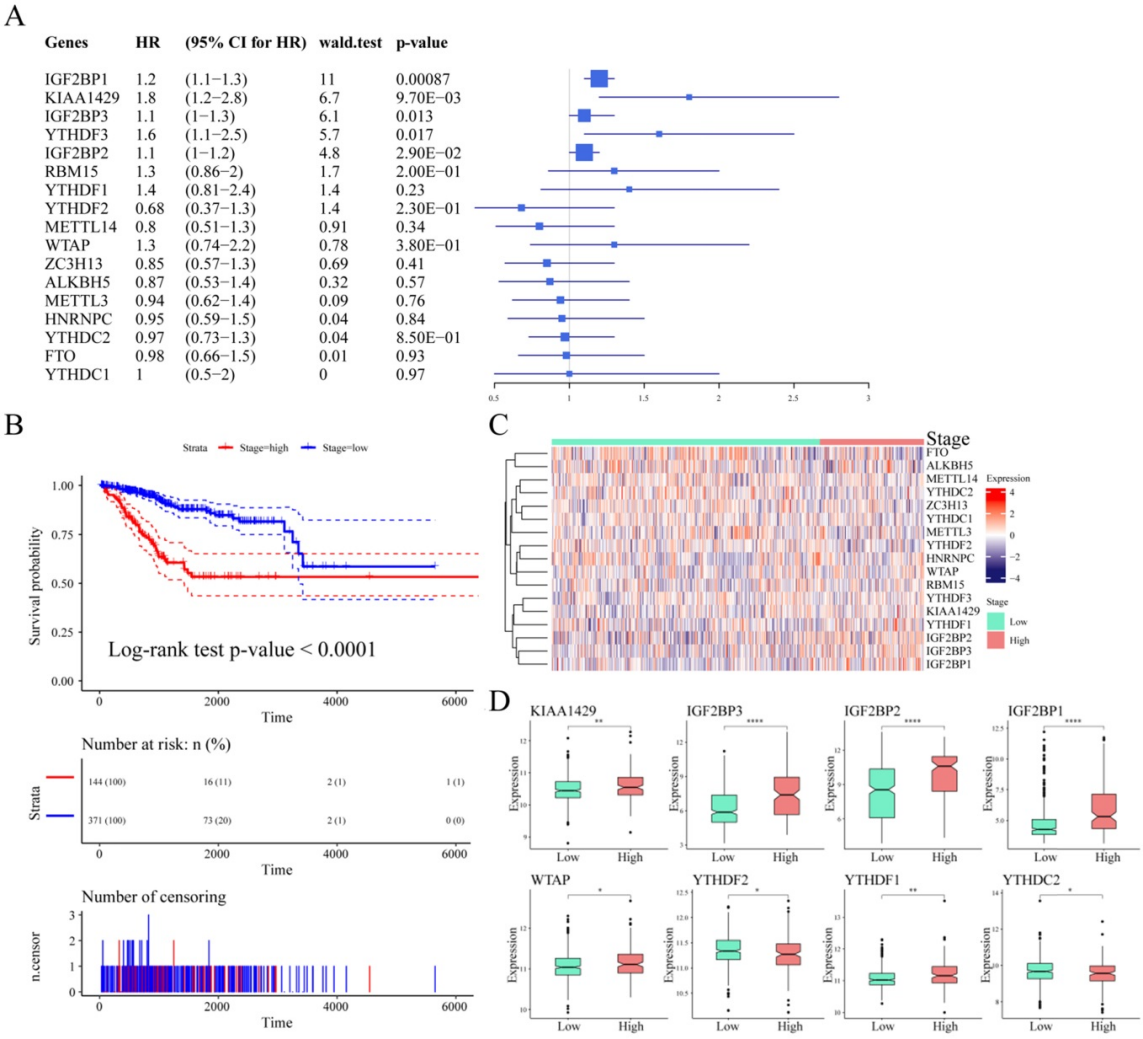
** Association between m6A regulatory genes and survival in UCEC patients.** (**A**) The Univariate Cox Analysis of m6A regulators in UCEC patients. (**B**) Kaplan-Meier curves of tumour stage and prognosis in patients. (**C**) The expression heatmap of m6A regulators in different tumour stages. (**D**) The expression levels of m6A regulatory genes in different stage cases.

To explore the prognostic value of m6A regulatory genes, we observed the relationship between the mRNA expression of these m6A genes and patient survival. As shown in **Table 4**, IGF2BP1, KIAA1429, IGF2BP3, YTHDF3, and IGF2BP2 were closely associated with UCEC patient survival outcomes (*p*-value < 0.05, **Figure 4A**). We observed a significant association between tumor stage and prognosis in UCEC (log-rank test *p*-value < 0.0001, **Figure 4B**). We considered stage I and stage II as low stage cases, while those above stage III were high stage cases. Based on this, the expression of m6A regulatory genes in different stage cases was clustered (**Figure 4C**). Next, we analyzed the differential expression of m6A regulatory genes in different clinical tumor stages. The results showed that although there was a significant association between clinical stage and patient survival, there was no significant relationship between the expression of 9 m6A regulatory genes and different stages. However, there were associations between the different stages and the expression of the other 8 m6A regulatory genes (**Figure 4D**).

**Figure 5:**
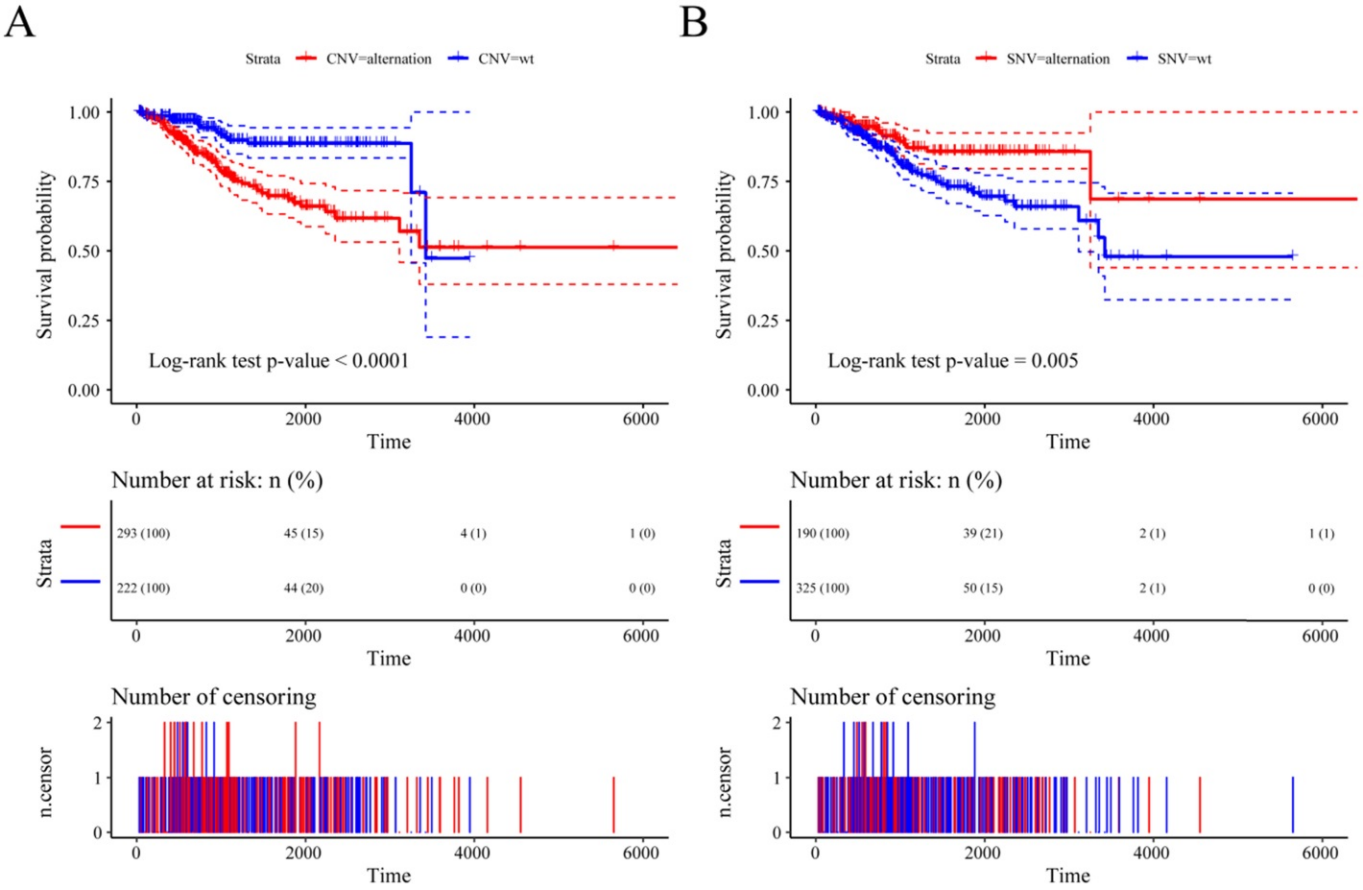
** The relationship between the CNV and SNV of m6A regulatory genes and UCEC patient survival.** (**A**) The relationship between the CNV of the m6A regulatory genes and patient survival. (**B**) The relationship between the SNV of the m6A regulatory genes and patient survival.

Previous studies have shown that CNV changes in m6A regulators can result in changes in m6A regulatory gene expression levels. Next, we used CNV as the research object to analyze the relationship between the CNV of m6A regulatory genes and UCEC patient survival. The results showed a significant relationship between the CNV of the m6A regulatory genes and patient survival (**Figure 5A**), and SNV was also significantly associated with patient survival (**Figure 5B**). At the same time, we used multivariate Cox regression to explore 17 m6A regulatory genes in the prognosis of patients. We used the expression of 17 m6A regulatory genes to assess patient risk and found that m6A regulatory gene expression can significantly predict patient risk (**Figure S1A**, **Table S3**). Furthermore, the AUC values at 1 year, 3 years, and 5 years were all larger than 0.65 (**Figure S1B**). The results indicated that the expression of m6A regulatory genes can be used as a prognostic marker for UCEC.

### A prognostic signature based on m6A regulatory genes

**Figure 6:**
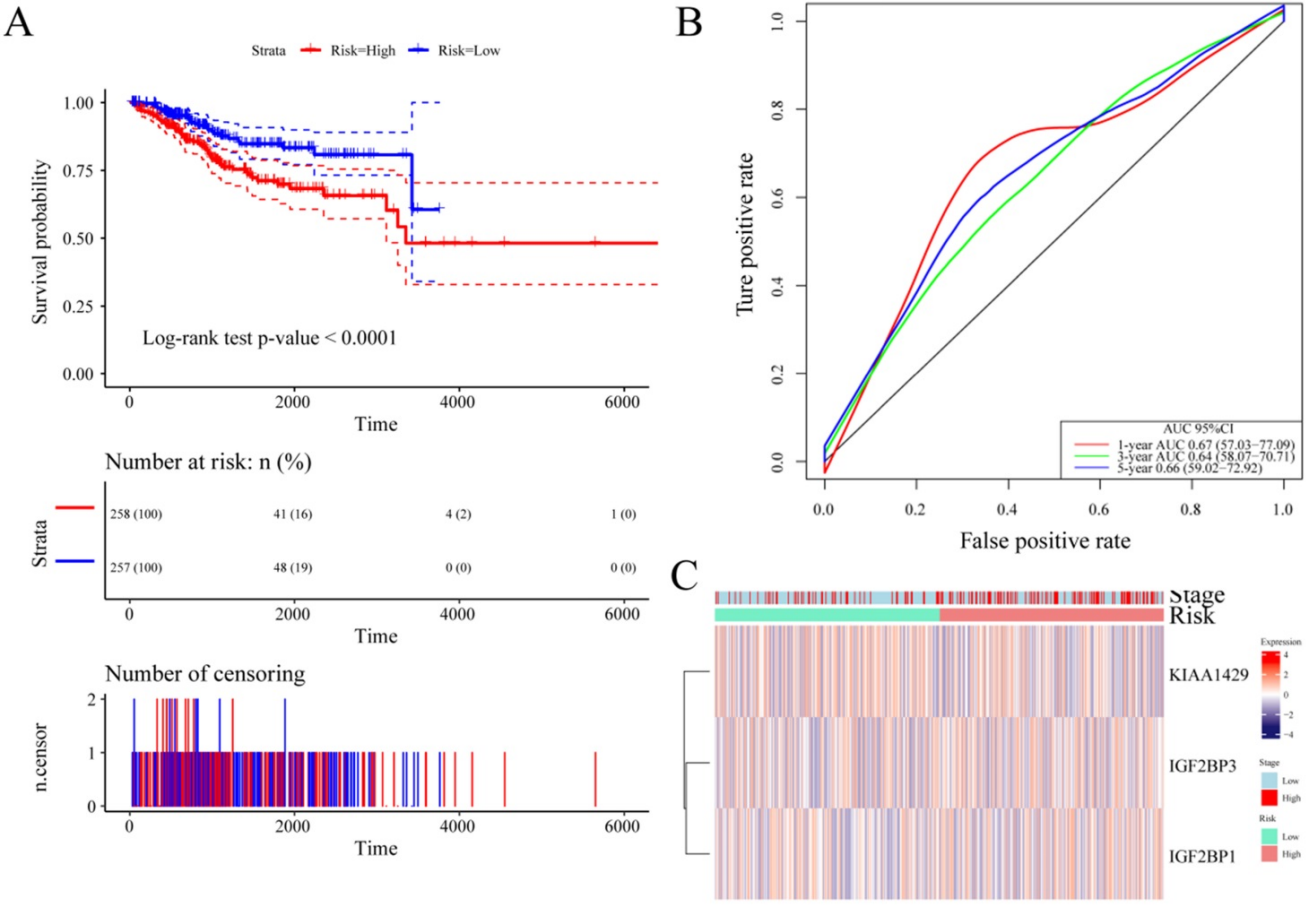
** Establishment of a prognostic signature based on m6A regulatory genes.** (**A**) Kaplan-Meier curves of the risk score and prognosis in UCEC patients. (**B**) The ROC of the prognostic signature based on m6A regulatory genes with 1-year, 3-year, and 5-year survival. (**C**) Expression heatmap of three genes in the two risk groups.

Based on the above results, to further reduce the number of prognostic markers, we performed LASSO analysis on the 17 m6A regulatory genes. Using 1,000 LASSO regressions, we found 3 genes that appeared in the LASSO results more than 100 times, namely, IGF2BP3, KIAA1429 and IGF2BP1 (**Table 5**). These genes cover two important m6A regulatory functions of “writers” and “readers”. Next, the expression of these three genes was used to predict UCEC patient risk scores. The formula was as follows: Risk score = (0.115 * expression value of IGF2BP1) + (0.399 * expression value of KIAA1429) + (0.052 * expression value of IGF2BP3). Using the median risk value to predict patient risk, it was found that the expression of these three genes can effectively predict UCEC patient risk (**Figure 6A**). The AUC values of the three m6A regulatory genes at 1 year, 3 years, and 5 years were all greater than 0.6 (**Figure 6B**), and the log-rank test *p*-value predicted by patients markers was also less than 0.0001. At the same time, we clustered the expression levels of these three m6A regulatory genes and their patient risk values and found that different genes predisposed patients to the high- and low-risk groups (**Figure 6C**).

Moreover, we focused on the impact of the expression of the above three genes on patient survival. The analysis found that the expression of three m6A regulatory genes was significantly different from the survival time of patients (**Figure S2**). The prognosis of patients with higher m6A regulatory gene expression levels was significantly worse than that of patients with lower expression levels. This result suggests that the expression levels of these three m6A regulatory genes have important clinical reference significance for UCEC patients.

### Association between prognostic signature and clinical pathology

**Figure 7:**
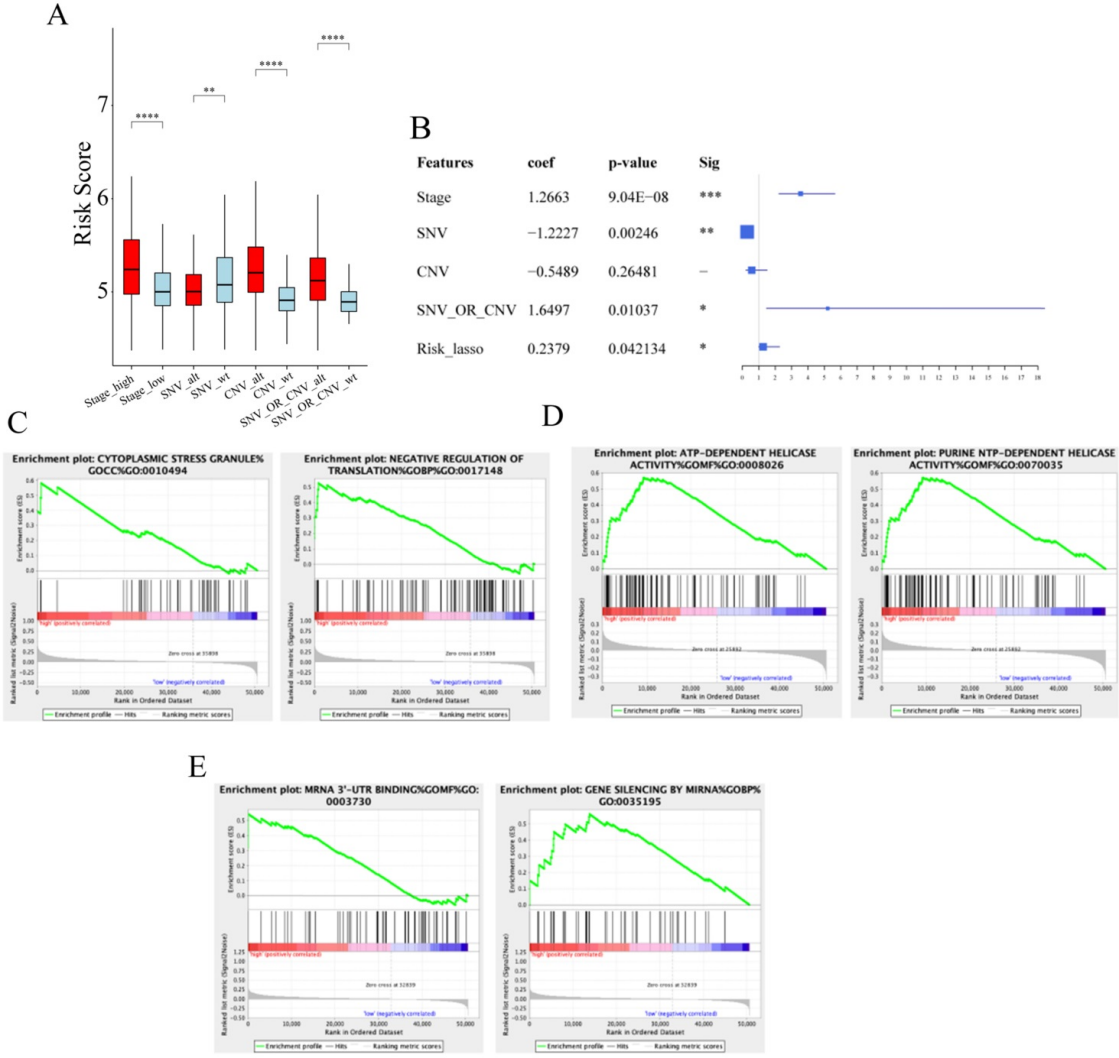
** The prognostic roles of risk score and functional enrichment analysis.** (**A**) The distribution of risk scores in tumour stages, CNV, and SNV. (**B**) The Univariate Cox Analysis of risk score in UCEC patients. (**C**) Enrichment results of IGF2BP1. (**D**) Enrichment results of KIAA1429. (**E**) Enrichment results of IGF2BP3.

We next examined whether there were associations between our prognostic signature and clinical pathology. As shown in **Figure 7A**, we observed that risk scores were significantly different between patients stratified by tumor stage, SNV, and CNV. Next, we performed multivariate Cox regression analyses to determine whether our risk signature is an independent prognostic indicator for UCEC. Multivariate Cox regression analysis suggested that the risk score might be an independent prognostic indicator for the overall survival of patients with UCEC (*p*-value < 0.05, **Figure 7B**).

### Functional enrichment analysis of IGF2BP1, KIAA1429 and IGF2BP3

Since IGF2BP1 and IGF2BP3 are “reader” genes in the m6A process and KIAA1429 is also an important gene in the m6A methylation process, we decided to investigate the role of m6A dysregulation in the pathogenesis of UCEC. We examined pathway enrichment with different expression levels of IGF2BP1, KIAA1429, and IGF2BP3. Gene enrichment analysis indicated that high IGF2BP1 gene expression is associated with important biological processes such as those involving cytoplasmic stress granules (**Figure 7C**). KIAA1429 gene expression is associated with cellular nucleic acid metabolism (**Figure 7D**). The expression of the IGF2BP3 gene is associated with processes such as RNA binding (**Figure 7E**, **Table S4**). This suggests a potential mechanism for the pathogenesis of UCEC.

## Discussion

In this study, we performed a multiomics study based on m6A regulators and built a prognostic gene signature of m6A regulators in UCEC. The genetic alterations in m6A regulatory genes could be effective and reliable biomarkers for UCEC prognosis prediction in the future.

m6A is the most prevalent internal RNA modification. Abnormal changes in m6A levels of regulators are closely related to the development of tumors 21. Increasing evidence indicates that m6A can regulate gene expression, which in turn regulates the cellular processes of cell self-renewal, differentiation, invasion, and apoptosis 10, 22. For example, METTL3, as an RNA m6A methyltransferase, can promote the growth of prostate cancer by regulating the Hedgehog pathway 23. This gene was also reported to be upregulated in melanoma and plays a role in invasion/migration through MMP2 24. In addition, METTL3 promotes osteosarcoma cell progression by regulating the m6A level of LEF1 and activating the Wnt/β-catenin signaling pathway 25. In colorectal cancer, compared with normal tissues, most m6A-related genes were significantly upregulated in tumor tissues, while METTL14, YTHDF3, and ALCBH5 were significantly downregulated in cancer tissues 26. In this study, survival analysis showed that the expression levels of METTL3, METTL14, METTL16, FTO and ALKBH5 were related to the clinical outcome of patients with CRC. In gastric cancer, Li *et al.* 27 reported that the abnormal expression of the demethylase genes FTO and ALKBH1 has obvious prognostic value in patients, suggesting that FTO and ALKBH1 may play an important role in progression and metastasis. These studies have shown the important roles of m6A regulators in human cancers.

CNV and SNV of m6A regulator genes are significantly associated with UCEC prognosis. The prognosis of patients with alterations in CNV was much poorer than that of those with no alterations. However, patients with alternations in SNV had a much better prognosis than patients with no alterations. If mutations occur, they are often harmful to genes. Mutations in m6A regulator genes may result in loss of their function. This also suggests that the normal m6A process plays an important role in the development of UCEC.

Recently, various prognostic risk signatures based on m6A regulators in human cancers were reported. For example, Chen *et al.* 28 used the gene expression profiles of bladder cancer from the TCGA database and established a risk signature including WTAP, YTHDC1 and FTO by using the LASSO Cox regression model. In their study, risk characteristics were not only independent prognostic markers of patients but also predictors of clinical pathological variables. Moreover, a two-gene prognostic signature including YTHDC2 and HNRNPC was constructed and could predict OS in head and neck squamous cell carcinoma patients from the TCGA database 13. In gastric cancer 15, using 3 m6A RNA methylation regulators (FTO, RBM15, ALKBH5), a prognostic risk signature was established. It is not only an independent prognostic marker but can also predict the clinicopathological features of gastric cancer. Additionally, based on the TCGA database, gene signatures and prognostic values of m6A regulators in clear cell renal cell carcinoma were identified 29. However, there has been no study regarding a risk signature of m6A regulators in UCEC, so we used previous research to support this research direction.

In our risk model, we identified a total of three m6A regulators, including IGF2BP1, KIAA1429 and IGF2BP3. First, IGF2BP1 (insulin-like growth factor 2 mRNA binding protein 1) plays significant roles in carcinogenesis, including tumor cell proliferation and growth, invasion, and chemoresistance, and is associated with poor overall survival and metastasis in various types of human cancers 30. This gene was also reported to be upregulated and associated with a poor prognosis in pancreatic cancer patients 31. It inhibits pancreatic cancer cell growth *in vitro* and *in vivo* via the AKT signaling pathway. In addition, miR-506 can inhibit proliferation and invasion by targeting IGF2BP1 in glioblastoma 32. KIAA1429 was shown to regulate the migration and invasion of hepatocellular carcinoma by modifying the m6A modification of ID2 mRNA 33. This gene can also act as an oncogenic factor in breast cancer 34. Moreover, its biological roles in liver cancer 35, head and neck squamous cell carcinoma 13, and lung cancer 36 were determined. Finally, IGF2BP3 plays significant biological roles in thyroid cancer 37, breast cancer 38, gastric cancer 39, and colorectal cancer 40. However, the above three m6A regulators have not been reported in UCEC.

Here, as **Figure 6C** shown, the 3 signature genes are indeed not correlated very well with stage or risk. However, there are a few questions to elaborate. First, we only show the expression of the above genes in different stages or risks here. Our primary goal is to look at the expression patterns of these genes in different stages or risks. Second, there was no significant association between these genes at different stages or risks. However, the risk score is related to different stages and risk groups, which also suggests that our prognostic signature of putting the above three genes together can distinguish different stages. In other words, the discriminative ability of the signature is stronger than the individual discriminative ability of each gene.

However, there are also some limitations in the present study. First, the robustness of our prognostic signature should be validated with a large sample size in future studies. Based on other public data or our own clinical samples, we should validate main findings and conclusions. Second, further experiments are required to validate the m6A regulators in this risk signature. Last, the AUC value of our prognostic signature was only 0.66 at 5 years. The major reason for this problem was as follows: In our study, the number of genes included is too small, but the focus of our study is only m6A regulators. Compared with other gene signature of UCEC, the genes they included are all human transcriptome or most other gene sets. Because of a greater reduction in the number of genes in our signature after the LASSO method, the AUC value may too low. Whether combined with other pathological factors or other significant genes can increase the value of AUC, it remains uncertain. In conclusion, we identified genetic alterations in m6A regulatory genes. These m6A RNA methylation regulators can participate in malignant progression. Thus, they could be effective and reliable biomarkers for UCEC prognosis prediction.

## Supplementary Material

Supplementary figure S1-2.

Supplementary table S1-4.

## Figures and Tables

**Table 1:** The CNV statistics of m6A regulatory genes in UCEC samples

Type	Genes	Diploid	Deletion	Amplification	CNV_sum	Amplification %	Deletion %	Percentage
Writers	METTL3	447	47	47	94	50.00%	50.00%	17.38%
	METTL14	456	78	8	86	9.30%	90.70%	15.87%
	WTAP	461	32	48	80	60.00%	40.00%	14.79%
	KIAA1429	374	11	156	167	93.41%	6.59%	30.87%
	RBM15	475	27	39	66	59.09%	40.91%	12.20%
	ZC3H13	431	87	24	111	21.62%	78.38%	20.48%
Readers	YTHDC1	474	65	28	93	30.11%	69.89%	16.40%
	YTHDC2	463	65	13	78	16.67%	83.33%	14.42%
	YTHDF3	386	10	145	155	93.55%	6.45%	28.65%
	YTHDF1	401	9	131	140	93.57%	6.43%	25.88%
	YTHDF2	445	62	30	92	32.61%	67.39%	17.13%
	HNRNPC	445	45	55	100	55.00%	45.00%	18.35%
	IGF2BP1	455	72	46	118	38.98%	61.02%	20.59%
	IGF2BP2	391	9	148	157	94.27%	5.73%	28.65%
	IGF2BP3	426	40	82	122	67.21%	32.79%	22.26%
Erasers	FTO	401	133	15	148	10.14%	89.86%	26.96%
	ALKBH5	396	121	28	149	18.79%	81.21%	27.34%
**Total**		**7,327**	**913**	**1,043**	**1,956**	**53.32%**	**46.68%**	**21.07%**

**Table 2:** The univariate Cox analysis of clinical characteristics and changes in m6A regulatory genes

Features	Beta	HR (95%CI for HR)	Wald.test	*p*-value
Stage	-1.4	0.24 (0.16-0.37)	43	6.90E-11
SNV	-0.72	0.49 (0.29-0.81)	7.6	6.00E-03
CNV	1.1	2.9 (1.7-4.9)	16	5.60E-05
CNV_or_SNV	1.3	3.5 (1.5-8.1)	8.8	3.00E-03

*Note* HR: Hazard Ratio, CI: Confidence Interval.

**Table 3:** Relationship between m6A regulatory gene alterations and UCEC-related biomarkers

Genes	Samples	Type	Without SNV and CNV	With SNV and CNV	*X^2^*	*p*-value
PTEN		wt	231	91	8.28651338	0.00399406
	n=542	alternation	311	72		
CTNNB1		wt	400	82	13.2480533	0.00027286
	n=542	alternation	142	7		
PIK3CA		wt	284	99	5.95330386	0.01468973
	n=542	alternation	258	56		
ARID1A		wt	307	92	45.3727938	1.6288E-11
	n=542	alternation	235	7		
KRAS		wt	418	85	0.31978025	0.57173974
	n=542	alternation	124	21		

**Table 4:** The univariate Cox analysis of m6A regulatory genes and survival

Genes	Beta	HR (95% CI for HR)	Wald.test	p-value
IGF2BP1	0.16	1.2 (1.1-1.3)	11	0.00087
KIAA1429	0.59	1.8 (1.2-2.8)	6.7	0.0097
IGF2BP3	0.14	1.1 (1-1.3)	6.1	0.013
YTHDF3	0.49	1.6 (1.1-2.5)	5.7	0.017
IGF2BP2	0.098	1.1 (1-1.2)	4.8	0.029
RBM15	0.28	1.3 (0.86-2)	1.7	0.2
YTHDF1	0.33	1.4 (0.81-2.4)	1.4	0.23
YTHDF2	-0.38	0.68 (0.37-1.3)	1.4	0.23
METTL14	-0.22	0.8 (0.51-1.3)	0.91	0.34
WTAP	0.25	1.3 (0.74-2.2)	0.78	0.38
ZC3H13	-0.17	0.85 (0.57-1.3)	0.69	0.41
ALKBH5	-0.14	0.87 (0.53-1.4)	0.32	0.57
METTL3	-0.064	0.94 (0.62-1.4)	0.09	0.76
HNRNPC	-0.05	0.95 (0.59-1.5)	0.04	0.84
YTHDC2	-0.028	0.97 (0.73-1.3)	0.04	0.85
FTO	-0.018	0.98 (0.66-1.5)	0.01	0.93
YTHDC1	0.013	1 (0.5-2)	0	0.97

*Note* HR: Hazard Ratio, CI: Confidence Interval.

**Table 5:** The LASSO analysis of m6A regulatory genes

Duplicates	Genes	Functions
871	IGF2BP1	Readers
682	KIAA1429	Writer
522	IGF2BP3	Readers
335	METTL14	Writer
204	YTHDF1	Readers
185	YTHDF2	Readers
126	YTHDF3	Readers
100	IGF2BP2	Readers
48	RBM15	Writer
47	ZC3H13	Writer
44	WTAP	Writer
32	METTL3	Writer
24	FTO	Erasers
10	YTHDC2	Readers
8	HNRNPC	Readers
7	ALKBH5	Erasers
4	YTHDC1	Readers
