# Supplementary figures and images for: Multiomics profile and prognostic gene signature of m6A regulators in uterine corpus endometrial carcinoma
Source: J Cancer. 2020 Sep 9;11(21):6390–401. doi: 10.7150/jca.46386 (PMC7532517; doi:10.7150/jca.46386)

A

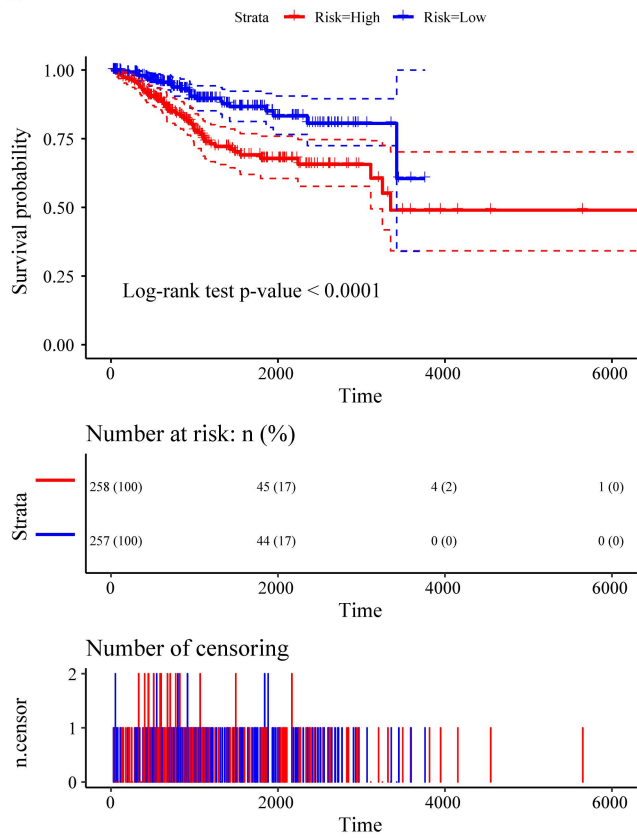

B

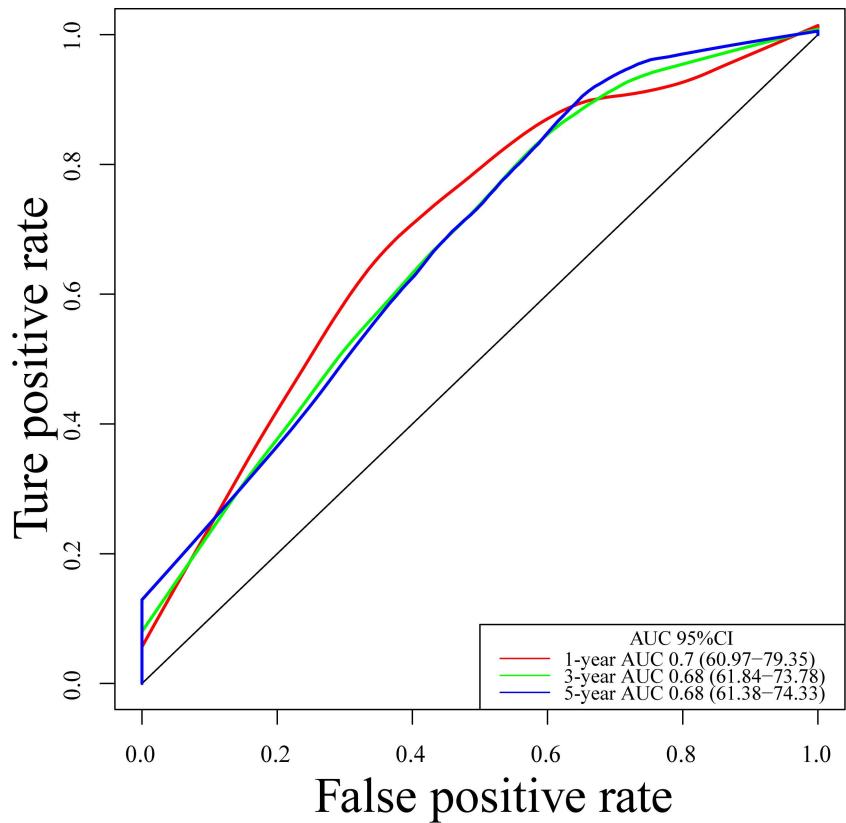

A

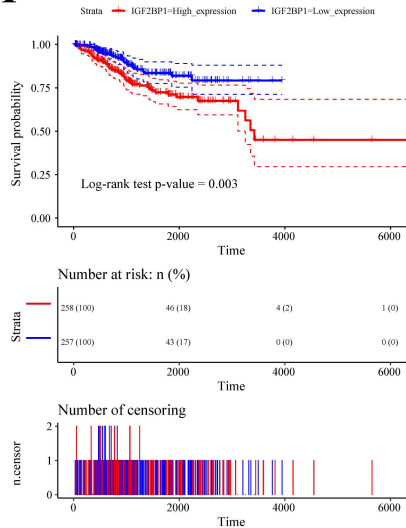

B

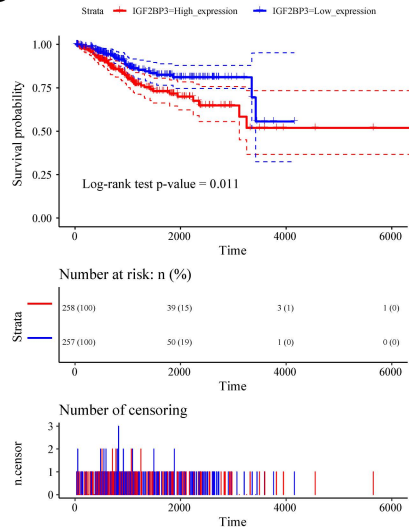

C

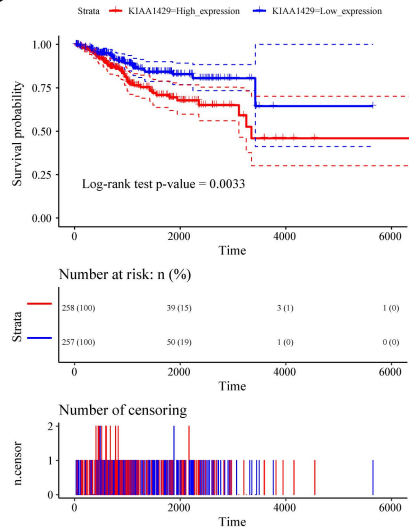

Supplement: Supplementary file 1 — Supplementary figure S1-2. [file jcav11p6390s1.pdf]
